# Supplementary material for: Atopic dermatitis and cognitive dysfunction in middle-aged and older adults: A systematic review and meta-analysis
Source: PLoS One. 2023 Oct 25;18(10):e0292987. doi: 10.1371/journal.pone.0292987 (PMC10599501; doi:10.1371/journal.pone.0292987)
Supplement: S2 Table — (DOCX) [file pone.0292987.s002.docx]

Supplementary Table 1. Search terms included for each library search

| **PUBMED**  ((((((((((((("Dermatitis, Atopic"[Mesh]) OR ("Atopic Dermatitides"[tiab])) OR ("Atopic Dermatitis"[tiab])) OR ("Dermatitides, Atopic"[tiab])) OR ("Neurodermatitis, Atopic"[tiab])) OR ("Atopic Neurodermatitides"[tiab])) OR ("Atopic Neurodermatitis"[tiab])) OR ("Neurodermatitides, Atopic"[tiab])) OR ("Disseminated Neurodermatitis"[tiab])) OR ("Eczema, Atopic"[tiab])) OR ("Atopic Eczema"[tiab])) OR ("Infantile Eczema"[tiab])) AND ((((((((((((((Dementia[Mesh]) OR (Dementias[tiab])) OR (Amentia[tiab])) OR (Amentias[tiab])) OR ("Senile Paranoid Dementia"[tiab])) OR ("Dementias, Senile Paranoid"[tiab])) OR ("Paranoid Dementia, Senile"[tiab])) OR ("Paranoid Dementias, Senile"[tiab])) OR ("Senile Paranoid Dementias"[tiab])) OR ("Familial Dementia"[tiab])) OR ("Dementias, Familial"[tiab])) OR (((((((((((((((("Alzheimer Disease"[Mesh]) OR ("Alzheimer Dementia"[tiab])) OR ("Alzheimer Dementias"[tiab])) OR ("Dementia, Alzheimer"[tiab])) OR ("Alzheimers Disease"[tiab])) OR ("Dementia, Senile"[tiab])) OR ("Senile Dementia"[tiab])) OR ("Dementia, Alzheimer Type"[tiab])) OR ("Alzheimer Type Dementia"[tiab])) OR ("Alzheimer-Type Dementia"(ATD[tiab]))) OR ("Alzheimer Syndrome"[tiab])) OR ("Alzheimers Diseases"[tiab])) OR ("Alzheimer Diseases"[tiab])) OR ("Alzheimers Diseases"[tiab])) OR ("Familial Alzheimer Disease"(FAD[tiab]))) OR ("Presenile Alzheimer Dementia"[tiab]))) OR (((((((("Dementia, Vascular"[Mesh]) OR ("Dementias, Vascular"[tiab])) OR ("Vascular Dementias"[tiab])) OR ("Vascular Dementia"[tiab])) OR ("Dementia, Subcortical Vascular"[tiab])) OR ("Arteriosclerotic Dementia"[tiab])) OR ("Binswanger Disease"[tiab])) OR ("Vascular Dementia, Subcortical"[tiab]))) OR (((((((("Mixed Dementias"[Mesh]) OR ("Dementia, Mixed"[tiab])) OR ("Mixed Dementia"[tiab])) OR ("Mixed-Etiology Dementias"[tiab])) OR ("Dementia, Mixed-Etiology"[tiab])) OR ("Mixed Etiology Dementias"[tiab])) OR ("Mixed-Etiology Dementia"[tiab])) OR ("Multiple-Etiology Dementias"[tiab])))) AND ((((((("Cohort Studies"[Mesh]) "Follow-Up Studies"[Mesh]) OR "Longitudinal Studies"[Mesh]) OR "Prospective Studies"[Mesh]) OR "Retrospective Studies"[Mesh]) OR "Cross-Sectional Studies"[Mesh]) OR ((((Cohort[tiab]) OR Cross-Sectional[tiab]) OR "Cross Sectional"[tiab]) OR Longitudinal[tiab]) AND (((elderly[tiab]) OR "old adults"[tiab])) OR ((Aged[Mesh]) OR "Middle Aged"[Mesh])) |
| --- |
| **EMBASE**  (((((((((((((exp "Dermatitis, Atopic"/) OR ("Atopic Dermatitides".tw.)) OR ("Atopic Dermatitis".tw.)) OR ("Dermatitides, Atopic".tw.)) OR ("Neurodermatitis, Atopic".tw.)) OR ("Atopic Neurodermatitides".tw.)) OR ("Atopic Neurodermatitis".tw.)) OR ("Neurodermatitides, Atopic".tw.)) OR ("Disseminated Neurodermatitis".tw.)) OR ("Eczema, Atopic".tw.)) OR ("Atopic Eczema".tw.)) OR ("Infantile Eczema".tw.)) AND ((((((((((((((exp Dementia/) OR (Dementias.tw.)) OR (Amentia.tw.)) OR (Amentias.tw.)) OR ("Senile Paranoid Dementia".tw.)) OR ("Dementias, Senile Paranoid".tw.)) OR ("Paranoid Dementia, Senile".tw.)) OR ("Paranoid Dementias, Senile".tw.)) OR ("Senile Paranoid Dementias".tw.)) OR ("Familial Dementia".tw.)) OR ("Dementias, Familial".tw.)) OR ((((((((((((((((exp "Alzheimer Disease"/) OR ("Alzheimer Dementia".tw.)) OR ("Alzheimer Dementias".tw.)) OR ("Dementia, Alzheimer".tw.)) OR ("Alzheimer's Disease".tw.)) OR ("Dementia, Senile".tw.)) OR ("Senile Dementia".tw.)) OR ("Dementia, Alzheimer Type".tw.)) OR ("Alzheimer Type Dementia".tw.)) OR ("Alzheimer-Type Dementia" (ATD.tw.))) OR ("Alzheimer Syndrome".tw.)) OR ("Alzheimer's Diseases".tw.)) OR ("Alzheimer Diseases".tw.)) OR ("Alzheimers Diseases".tw.)) OR ("Familial Alzheimer Disease" (FAD.tw.))) OR ("Presenile Alzheimer Dementia".tw.))) OR ((((((((exp "Dementia, Vascular"/) OR ("Dementias, Vascular".tw.)) OR ("Vascular Dementias".tw.)) OR ("Vascular Dementia".tw.)) OR ("Dementia, Subcortical Vascular".tw.)) OR ("Arteriosclerotic Dementia".tw.)) OR ("Binswanger Disease".tw.)) OR ("Vascular Dementia, Subcortical".tw.))) OR ((((((((exp "Mixed Dementias"/) OR ("Dementia, Mixed".tw.)) OR ("Mixed Dementia".tw.)) OR ("Mixed-Etiology Dementias".tw.)) OR ("Dementia, Mixed-Etiology".tw.)) OR ("Mixed Etiology Dementias".tw.)) OR ("Mixed-Etiology Dementia".tw.)) OR ("Multiple-Etiology Dementias".tw.)))) AND (((((((exp "Cohort Studies"/) exp "Follow-Up Studies"/) OR exp "Longitudinal Studies"/) OR exp "Prospective Studies"/) OR exp "Retrospective Studies"/) OR exp "Cross-Sectional Studies"/) OR ((((Cohort.tw.) OR Cross-Sectional.tw.) OR "Cross Sectional".tw.) OR Longitudinal.tw.) AND (((elderly.tw.) OR "old adults".tw.)) OR ((exp Aged/) OR exp "Middle Aged"/)) |
| **WEB OF SCIENCE**  ((((((((((((("Dermatitis, Atopic") OR ("Atopic Dermatitides")) OR ("Atopic Dermatitis")) OR ("Dermatitides, Atopic")) OR ("Neurodermatitis, Atopic")) OR ("Atopic Neurodermatitides")) OR ("Atopic Neurodermatitis")) OR ("Neurodermatitides, Atopic")) OR ("Disseminated Neurodermatitis")) OR ("Eczema, Atopic")) OR ("Atopic Eczema")) OR ("Infantile Eczema")) AND ((((((((((((((Dementia) OR (Dementias)) OR (Amentia)) OR (Amentias)) OR ("Senile Paranoid Dementia")) OR ("Dementias, Senile Paranoid")) OR ("Paranoid Dementia, Senile")) OR ("Paranoid Dementias, Senile")) OR ("Senile Paranoid Dementias")) OR ("Familial Dementia")) OR ("Dementias, Familial")) OR (((((((((((((((("Alzheimer Disease") OR ("Alzheimer Dementia")) OR ("Alzheimer Dementias")) OR ("Dementia, Alzheimer")) OR ("Alzheimers Disease")) OR ("Dementia, Senile")) OR ("Senile Dementia")) OR ("Dementia, Alzheimer Type")) OR ("Alzheimer Type Dementia")) OR ("Alzheimer-Type Dementia"(ATD))) OR ("Alzheimer Syndrome")) OR ("Alzheimers Diseases")) OR ("Alzheimer Diseases")) OR ("Alzheimers Diseases")) OR ("Familial Alzheimer Disease"(FAD))) OR ("Presenile Alzheimer Dementia"))) OR (((((((("Dementia, Vascular") OR ("Dementias, Vascular")) OR ("Vascular Dementias")) OR ("Vascular Dementia")) OR ("Dementia, Subcortical Vascular")) OR ("Arteriosclerotic Dementia")) OR ("Binswanger Disease")) OR ("Vascular Dementia, Subcortical"))) OR (((((((("Mixed Dementias") OR ("Dementia, Mixed")) OR ("Mixed Dementia")) OR ("Mixed-Etiology Dementias")) OR ("Dementia, Mixed-Etiology")) OR ("Mixed Etiology Dementias")) OR ("Mixed-Etiology Dementia")) OR ("Multiple-Etiology Dementias")))) AND ((((((("Cohort Studies") "Follow-Up Studies") OR "Longitudinal Studies") OR "Prospective Studies") OR "Retrospective Studies") OR "Cross-Sectional Studies") OR ((((Cohort) OR Cross-Sectional) OR "Cross Sectional") OR Longitudinal) AND (((elderly) OR "old adults")) OR ((Aged) OR "Middle Aged")) |
