# Supplementary material for: Atopic dermatitis and cognitive dysfunction in middle-aged and older adults: A systematic review and meta-analysis
Source: PLoS One. 2023 Oct 25;18(10):e0292987. doi: 10.1371/journal.pone.0292987 (PMC10599501; doi:10.1371/journal.pone.0292987)
Supplement: S3 Table — (DOCX) [file pone.0292987.s003.docx]

| **Supplementary Table 3. Overview of Studies on the Associations between Atopic Dermatitis** **and Cognitive Outcomes in Included Studies** | | | | | | | | | | | | | |
| --- | --- | --- | --- | --- | --- | --- | --- | --- | --- | --- | --- | --- | --- |
| Author, year | Baseline Study Years | Country or region | Population | No.Of Participants/ Cases | prevalence of atopic dermatitis(%) | Gender (% Female) | Study design | Setting | Follow-up (Years) | Mean Age (SD) | Adjustment for Potential Confounders | Type of Cognitive Dysfunction | Outcome(HR,95% CI) |
| Johet al.,(2023A^)^**^1^** | 2009- 2017 | Korean | National Health Information Database | 6,785,948/260,705 | 0.4 | 49.9 | Prospective cohort study | Community residents | 8.1± 1.2 | 54.4± 10.5 | Age (continuous) and sex.for body mass index (<18.5, 18.5–22.9, 23–24.9, ≥25 kg/m2), smoking status (never, past, current), alcohol use (none, moderate, heavy), regular physical activity (yes, no), income (quartile), and area of residence (urban, rural), history of hypertension, dyslipidemia, diabetes, stroke, and myocardial infarction, systolic blood pressure, glucose ,high-density lipoprotein cholesterol , low-density lipoprotein cholesterol, and estimated glomerular filtration rate | All-cause dementia | HR1.16 (1.11–1.21) |
| Joh et al.,(2023B)**^1^** | 2009- 2017 | Korean | National Health Information Database | 6,785,948/195,739 | 0.4 | 49.9 | Prospective cohort study | Community residents | 8.1± 1.2 | 54.4± 10.5 | Age (continuous) and sex.for body mass index (<18.5, 18.5–22.9, 23–24.9, ≥25 kg/m2), smoking status (never, past, current), alcohol use (none, moderate, heavy), regular physical activity (yes, no), income (quartile), and area of residence (urban, rural), history of hypertension, dyslipidemia, diabetes, stroke, and myocardial infarction, systolic blood pressure, glucose ,high-density lipoprotein cholesterol , low-density lipoprotein cholesterol, and estimated glomerular filtration rate | Alzheimer disease dementia | HR 1.15 (1.09–1.21) |
| Joh et al.,(2023C)**^1^** | 2009- 2017 | Korean | National Health Information Database | 6,785,948/ 32,789 | 0.4 | 49.9 | Prospective cohort study | Community residents | 8.1± 1.2 | 54.4± 10.5 | Age (continuous) and sex.for body mass index (<18.5, 18.5–22.9, 23–24.9, ≥25 kg/m2), smoking status (never, past, current), alcohol use (none, moderate, heavy), regular physical activity (yes, no), income (quartile), and area of residence (urban, rural), history of hypertension, dyslipidemia, diabetes, stroke, and myocardial infarction, systolic blood pressure, glucose ,high-density lipoprotein cholesterol , low-density lipoprotein cholesterol, and estimated glomerular filtration rate | Vascular dementia | HR1.18 (1.04–1.34) |
| Magyari et al.,(2022A)**^2^** | - | United Kingdom | The Health Improvement Network | 1,767,667/ 57,263 | 12 | - | Prospective cohort study | Community residents | - | 》60 | Sex, calendar period, practice, Townsend score,  history of smoking, history of alcohol use, and body mass index. depression, anxiety, diabetes,hypertension, major adverse cardiovascular events, asthma,  rhinitis, and antihistamine prescriptions. | All-cause dementia | HR1.19 (1.16-1.22) |
| Magyari et al.,(2022B)**^2^** | - | United Kingdom | The Health Improvement Network | 1,767,667/ 57,263 | 12 | - | Prospective cohort study | Community residents | - | 》60 | Age, sex, and socioeconomic status | Alzheimer disease dementia | HR1.69 (1.61-1.78) |
| Magyari et al.,(2022C)**^2^** | - | United Kingdom | The Health Improvement Network | 1,767,667/ 57,263 | 12 | - | Prospective cohort study | Community residents | - | 》60 | Age, sex, and socioeconomic status | Vascular dementia | HR1.88 (1.78-1.99) |
| Eriksson et al.,(2008A)**^3^** | 1998 -2001 | Swedish | Swedish Twin Registry | 7,800/303 | 9.0 | - | Cross-Sectional Study | Community residents | - | 74.1± 6.8 | History of smoking, level of education and myocardial infarction | All-cause dementia | HR0.82 (0.58–1.17) |
| Eriksson et al.,(2008B)**^3^** | 1998 -2001 | Swedish | Swedish Twin Registry | 7,800/303 | 9.0 | - | Cross-Sectional Study | Community residents | - | 74.1± 6.8 | History of smoking, level of education and myocardial infarction | Alzheimer disease dementia | HR 0.92 (0.60–1.41) |
| Eriksson et al.,(2008C)**^3^** | 1974- 2001 | Swedish | Swedish Twin Registry | 22,188/1,332 | 9.0 | - | Prospective cohort study | Community residents | 22.6 ± 8 | 52.9 (37.0–71.0) | Age, sex, history of smoking, level of education  and myocardial infarction | All-cause dementia | HR 1.06(0.86–1.29) |
| Eriksson et al.,(2008D)**^3^** | 1974- 2001 | Swedish | Swedish Twin Registry | 22,188/  1,332 | 9.0 | - | Prospective cohort study | Community residents | 22.6 ± 8 | 52.9 (37.0–71.0) | Age, sex, history of smoking, level of education  and myocardial infarction | Alzheimer disease dementia | HR1.19(0.95–1.51) |
| Pan, et al.,(2021A)**^4^** | 1996-2013 | Taiwan | National Health Insurance Research Database | 11,649/86 | - | 51.1 | Prospective cohort study | Community residents | - | 》45 | Demographic data, comorbidities, CCI scores, and allcause clinical visits. | All-cause dementia | HR 2.02(1.24-3.29) |
| Pan, et al.,(2021B)**^4^** | 1996-2013 | Taiwan | National Health Insurance Research Database | 11,649/15 | - | 51.1 | Prospective cohort study | Community residents | - | 》45 | Demographic data, comorbidities, CCI scores, and allcause clinical visits | Alzheimer disease dementia | HR 3.74(1.17-11.97) |
| Shang, et al.,(2021)**^5^** | 2006−2010 | United Kingdom | UK Biobank | 471,485/ 6,189 | 1.2 | 54.5 | Retrospective cohort study | Community residents | 11.9 (11.2−12.6) | 56.8 ±8.0 | Age and gender,s education, income, BMI, smoking, physical activity, alcohol consumption, sleep duration, and diet | All-cause dementia | HR 1.16(1.02−1.31) |
| Shang, et al.,(2021)**^5^** | 2006−2010 | United Kingdom | UK Biobank | 471,485/ 2,597 | 0.5 | 54.5 | Retrospective cohort study | Community residents | 11.9 (11.2−12.6) | 56.8 ±8.0 | Age and gender,s education, income, BMI, smoking, physical activity, alcohol consumption, sleep duration, and diet | Alzheimer disease dementia | HR 1·18 (0·98-1·43) |
| Shang, et al.,(2021)**^5^** | 2006−2010 | United Kingdom | UK Biobank | 471,485/ 1,381 | 0·30 | 54.5 | Retrospective cohort study | Community residents | 11.9 (11.2−12.6) | 56.8 ±8.0 | Age and gender,s education, income, BMI, smoking, physical activity, alcohol consumption, sleep duration, and diet | Vascular dementia | HR 1.26 (0.98-1.62) |

**References**

1. Joh HK, Kwon H, Son KY, Yun JM, Cho SH, Han K, Park JH, Cho B. Allergic Diseases and Risk of Incident Dementia and Alzheimer's Disease. Ann Neurol. 2023 Feb;93(2):384-397. PMID: 36093572.
2. Magyari A, Ye M, Margolis DJ, McCulloch CE, Cummings SR, Yaffe K, Langan SM, Abuabara K. Adult atopic eczema and the risk of dementia: A population-based cohort study. J Am Acad Dermatol. 2022 Aug;87(2):314-322. Epub 2022 Mar 31. PMID: 35367295.
3. Eriksson UK, Gatz M, Dickman PW, Fratiglioni L, Pedersen NL. Asthma, eczema, rhinitis and the risk for dementia. Dement Geriatr Cogn Disord. 2008;25(2):148-56. PMID: 18097143.
4. Pan TL, Bai YM, Cheng CM, Tsai SJ, Tsai CF, Su TP, Li CT, Lin WC, Chen TJ, Liang CS, Chen MH. Atopic dermatitis and dementia risk: A nationwide longitudinal study. Ann Allergy Asthma Immunol. 2021 Aug;127(2):200-205.PMID: 33716147.
5. Shang X, Zhu Z, Zhang X, et al. Association of a wide range of chronic diseases and apolipoprotein E4 genotype with subsequent risk of dementia in community-dwelling adults: A retrospective cohort study[J]. EClinicalMedicine, 2022, 45.
