# Supplementary material for: Atopic dermatitis and cognitive dysfunction in middle-aged and older adults: A systematic review and meta-analysis
Source: PLoS One. 2023 Oct 25;18(10):e0292987. doi: 10.1371/journal.pone.0292987 (PMC10599501; doi:10.1371/journal.pone.0292987)
Supplement: S4 Table — (DOCX) [file pone.0292987.s004.docx]

| **S4 Table. Assessment of study quality** | | | | | | | | | | |
| --- | --- | --- | --- | --- | --- | --- | --- | --- | --- | --- |
|  | **Selection** | | | | **Comparability** | |  | **Outcome** |  |  |
| **Study** | **Representative of the exposed cohort** | **Selection of external control** | **Ascertainment of exposure** | **Outcome of interest not present at the start of the study** | **Main factor** | **Additional factor** | **Assessment of outcomes** | **Sufficient follow-up time** | **Adequacy of follow-up** | **Total** |
| Joh et al.,(2023) | 0 | 0 | 1 | 1 | 1 | 1 | 1 | 0 | 1 | 6 |
| Magyari et al.,(2022) | 1 | 1 | 1 | 1 | 1 | 1 | 1 | 1 | 1 | 9 |
| Eriksson et al.,(2008) | 1 | 1 | 1 | 1 | 1 | 1 | 1 | 1 | 0 | 8 |
| Pan et al.,(2021) | 1 | 1 | 0 | 1 | 0 | 1 | 1 | 1 | 1 | 7 |
| Shang et al.,(2021) | 1 | 0 | 1 | 1 | 0 | 1 | 1 | 1 | 1 | 7 |

References

1.Joh HK, Kwon H, Son KY, Yun JM, Cho SH, Han K, Park JH, Cho B. Allergic Diseases and Risk of Incident Dementia and Alzheimer's Disease. Ann Neurol. 2023 Feb;93(2):384-397. PMID: 36093572.

2.Magyari A, Ye M, Margolis DJ, McCulloch CE, Cummings SR, Yaffe K, Langan SM, Abuabara K. Adult atopic eczema and the risk of dementia: A population-based cohort study. J Am Acad Dermatol. 2022 Aug;87(2):314-322. Epub 2022 Mar 31. PMID: 35367295.

3.Eriksson UK, Gatz M, Dickman PW, Fratiglioni L, Pedersen NL. Asthma, eczema, rhinitis and the risk for dementia. Dement Geriatr Cogn Disord. 2008;25(2):148-56. PMID: 18097143.

4.Pan TL, Bai YM, Cheng CM, Tsai SJ, Tsai CF, Su TP, Li CT, Lin WC, Chen TJ, Liang CS, Chen MH. Atopic dermatitis and dementia risk: A nationwide longitudinal study. Ann Allergy Asthma Immunol. 2021 Aug;127(2):200-205.PMID: 33716147.

5.Shang X, Zhu Z, Huang Y, Zhang X, Wang W, Shi D, Jiang Y, Yang X, He M. Associations of ophthalmic and systemic conditions with incident dementia in the UK Biobank. Br J Ophthalmol. 2023 Feb;107(2):275-282. PMID: 34518160.
