# Supplementary figures and images for: Atopic dermatitis and cognitive dysfunction in middle-aged and older adults: A systematic review and meta-analysis
Source: PLoS One. 2023 Oct 25;18(10):e0292987. doi: 10.1371/journal.pone.0292987 (PMC10599501; doi:10.1371/journal.pone.0292987)

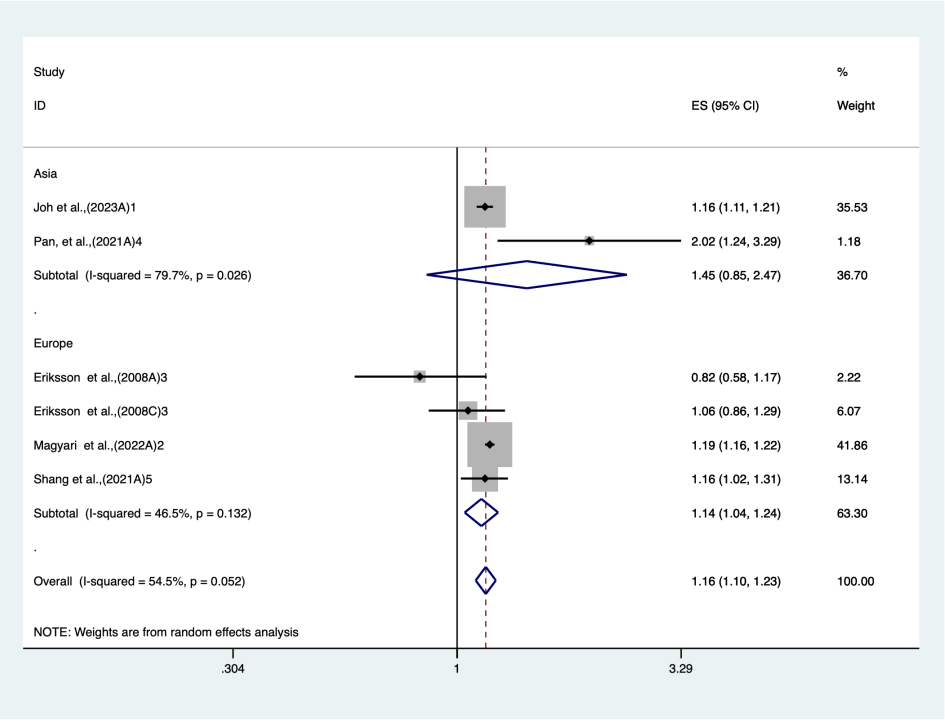

Supplement: S1 Fig — (TIF) [file pone.0292987.s005.tif]

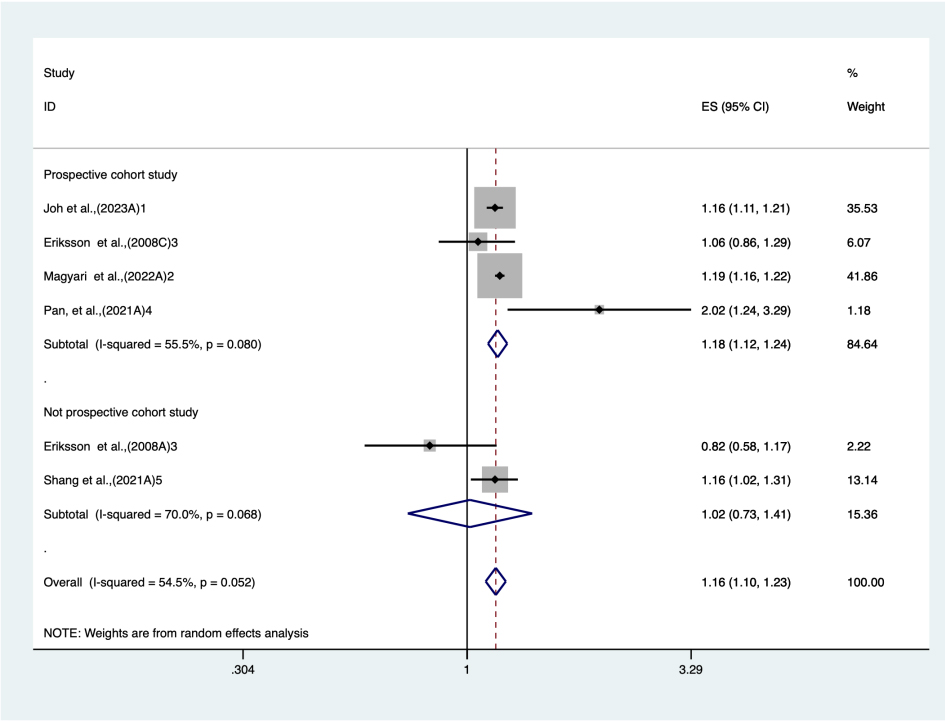

Supplement: S2 Fig — (TIF) [file pone.0292987.s006.tif]

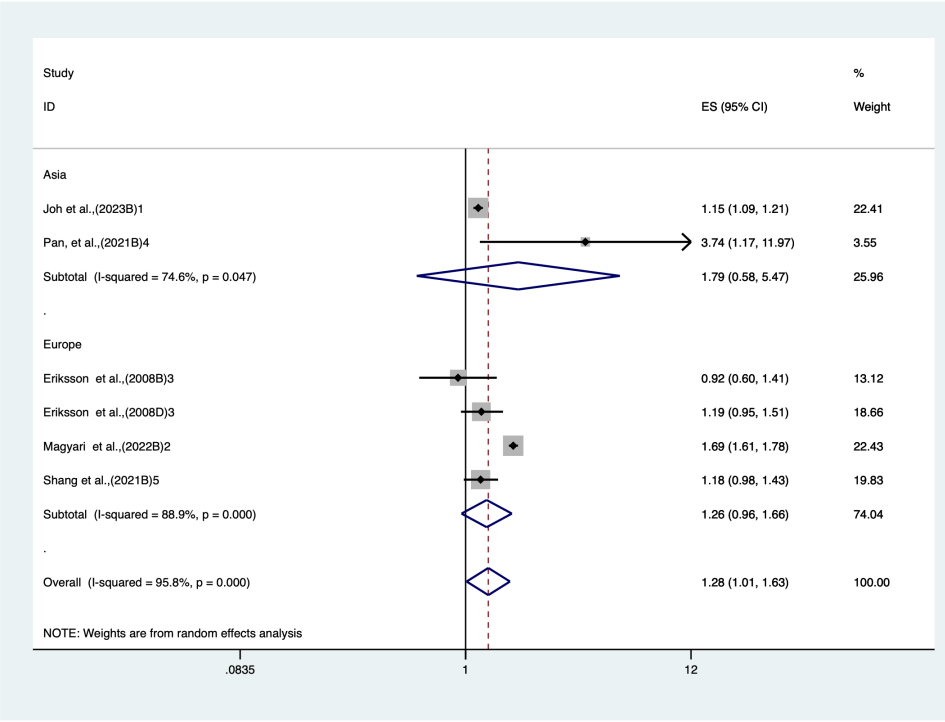

Supplement: S3 Fig — (TIF) [file pone.0292987.s007.tif]

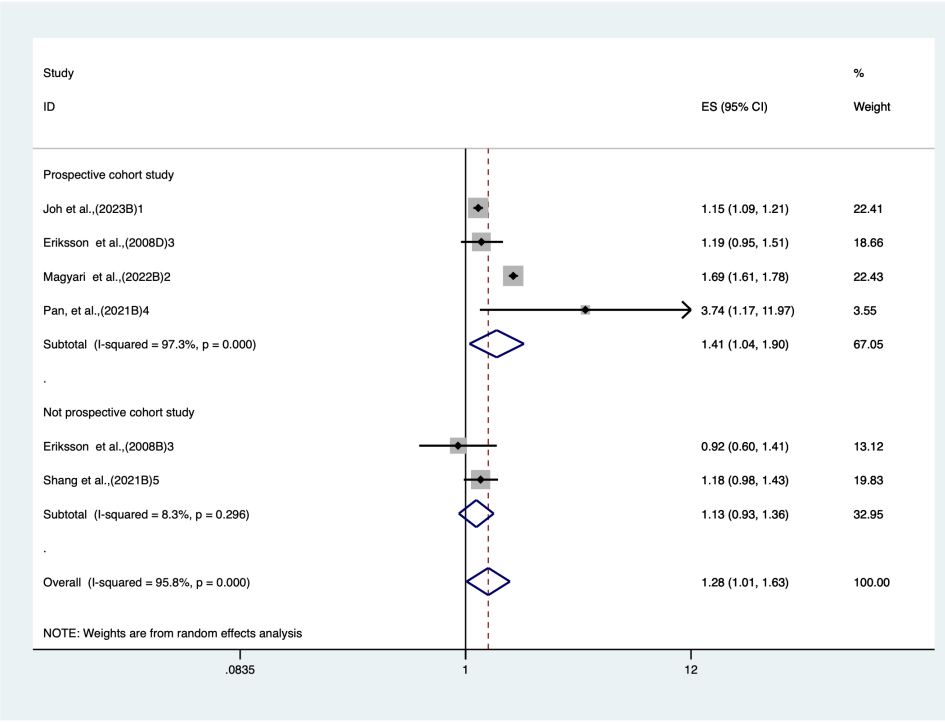

Supplement: S4 Fig — (TIF) [file pone.0292987.s008.tif]

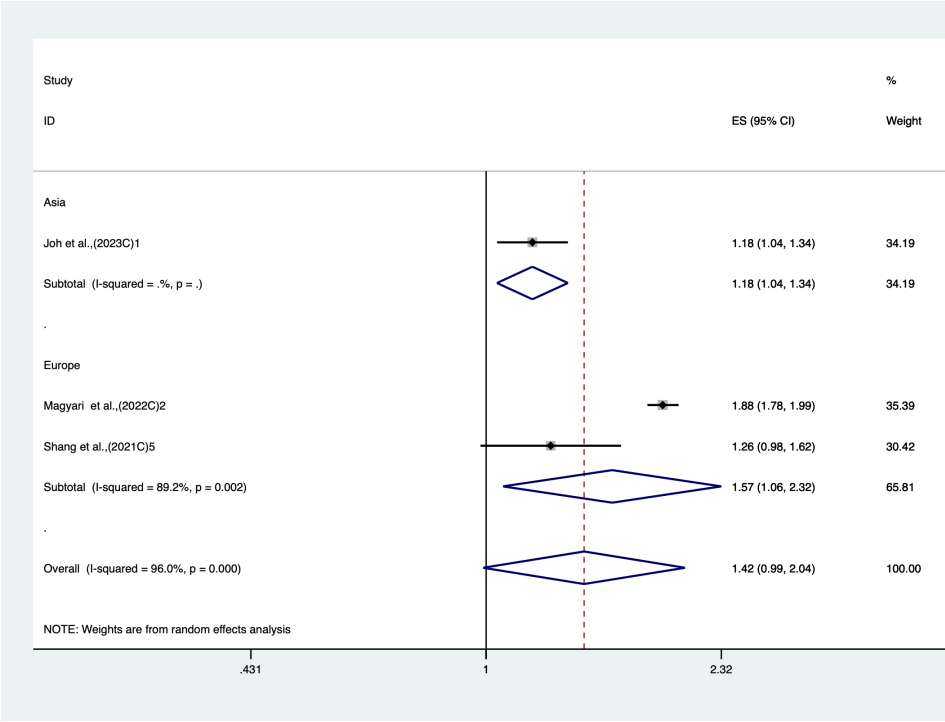

Supplement: S5 Fig — (TIF) [file pone.0292987.s009.tif]

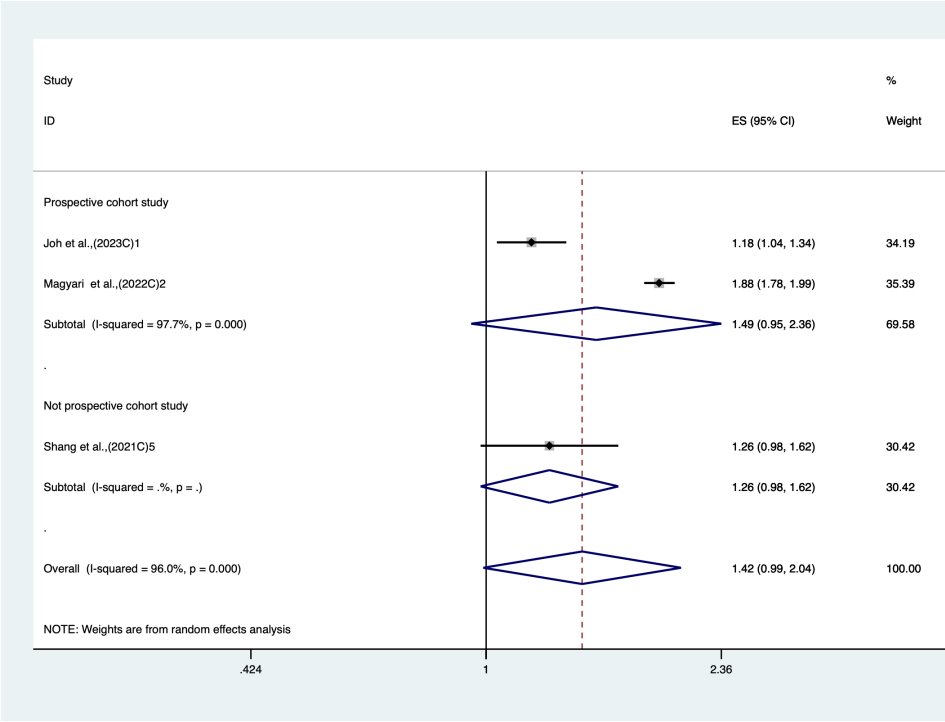

Supplement: S6 Fig — (TIF) [file pone.0292987.s010.tif]
