# Supplementary material for: Atopic dermatitis and cognitive dysfunction in middle-aged and older adults: A systematic review and meta-analysis
Source: PLoS One. 2023 Oct 25;18(10):e0292987. doi: 10.1371/journal.pone.0292987 (PMC10599501; doi:10.1371/journal.pone.0292987)
Supplement: Attachment — Submitted filename: Response to Reviewers.docx [file pone.0292987.s011.docx]

Dear editor and reviewers,

Thank you for your letter dated July 12, 2023. On behalf of my colleagues, I am herewith submitting the revised manuscript (PONE-D-23-12015) entitled “Atopic dermatitis and cognitive dysfunction in middle-aged and older adults: A systematic review and meta-analysis” for consideration of publication in Plos one. We would like to thank the editor and reviewers’ work devoted to our manuscript and we are very grateful for their valuable suggestions. We have considered the comments carefully and have made revisions (highlighted in red in the revised manuscript with track changes ) which we hope meet with approval.

Journal requirements:

1.Please ensure that your manuscript meets PLOS ONE's style requirements, including those for file naming.

Response: Reviewed and revised as suggested.

2. We note that you have stated that you will provide repository information for your data at acceptance. Should your manuscript be accepted for publication, we will hold it until you provide the relevant accession numbers or DOIs necessary to access your data. If you wish to make changes to your Data Availability statement, please describe these changes in your cover letter and we will update your Data Availability statement to reflect the information you provide.

Response: Thank you for your email and your interest in our manuscript. We appreciate the opportunity to revise our Data Availability statement according to the Journal requirements. We have made the following changes to our statement:We have removed the sentence that stated that we will provide repository information at acceptance, as this is no longer applicable.and all relevant data are within the manuscript and its Supporting Information files.

3. Please upload a new copy of Figure 3 as the detail is not clear.

Response: Thank you for your feedback on our manuscript. We have uploaded a new version of Figure 3 with higher resolution and contrast to improve the clarity of the details. We have also updated the caption and the text in the manuscript to reflect the changes in the figure. We hope that this revision meets your expectations and enhances the quality of our paper. Please let us know if you have any further comments or questions.

Comments to the Author:

Reviewer #1: This study presents novel perspectives regarding the association between atopic dermatitis and cognitive impairment. Unfortunately, the methodology of this study raised several concerns.

1.The inclusion criteria were poorly defined ie. cognitive dysfunction, middle-aged and older adults.

Response: We agree that the inclusion criteria were not clearly specified in the original manuscript. We have now added more details on how we defined cognitive dysfunction and middle-aged and older adults in the Methods section. Specifically, we used the following definitions:

Cognitive dysfunction: any impairment in cognitive domains such as memory, attention, executive function, language, or visuospatial skills, as measured by validated cognitive tests, a physician's diagnosis, or a self-reported measure.

Middle-aged and older adults: According to the World Health Organization (WHO), middle-aged adults are those between 45 and 59 years old, while older adults are those aged 60 years and above.

We hope that these clarifications will improve the quality and validity of our study.

2. Why did studies reporting outcomes other than the cognitive incidence excluded?

Response: Thank you for your reply. We appreciate your careful reading of our manuscript and your suggestion to improve the clarity of our exclusion criterion. We agree that the word "incidence" is redundant in this sentence and we have deleted it. The revised sentence now reads: "Studies were excluded if they: (1) reported outcomes other than the risk of cognitive dysfunction in the middle-aged and older adults; ..." We hope that this change addresses your concern and improves the quality of our manuscript.

3. The authors aimed to include case-control studies but specified that eligible studies must report the incidence of cognitive dysfunction. Case-control studies are not designed for studying incidence.

Response:Thank you for your valuable comments and suggestions. We appreciate your interest in our study and your constructive feedback. We have revised our manuscript accordingly and addressed each point as follows:

We apologize for the confusion caused by our use of the term "incidence". We agree that case-control studies are not suitable for estimating incidence rates. However, we did not intend to calculate the incidence rates of cognitive dysfunction in our study population. Rather, we used the term "incidence" to refer to the risk of cognitive dysfunction as an outcome variable in the included studies. We have clarified this point in the Methods section and replaced the term "incidence" with "risk" throughout the manuscript. We hope this change resolves your concern.

4. The purpose of conducting a systematic review is to identify and comprehensively synthesize all the relevant studies. Excluding studies with a Newcastle-Ottawa score of less than 4 might be considered inappropriate in the stage of the selection process. To avoid the effect of low-quality studies, the authors can perform sensitivity analysis instead of excluding the studies right from the beginning.

Response:Thank you for your valuable comments and suggestions. We appreciate your interest in our study and your constructive feedback. We agree with your point that excluding studies with a low Newcastle-Ottawa score might not be the best approach to conduct a systematic review and meta-analyses. We have revised our inclusion and exclusion criteria accordingly,and because we ultimately verified the papers we included and excluded did not involve studies with a NOS score of less than 4, there was no special impact on the subsequent research results.We hope that these changes address your concerns and improve the quality of our manuscript.

5. The authors did not specify the statistical model for the meta-analysis ie. DerSimonian and Laird or the weight model for the meta-analysis ie. inverse variance.

Response:Thank you for your comment. We appreciate your interest in our study and your constructive feedback. We agree that we should specify the statistical model and the weight model for the meta-analysis, as they may affect the results and interpretation of our findings. Therefore, we have revised our manuscript to include these details and explain our rationale for choosing them. We hope that these changes address your concern and improve the quality of our manuscript.

6. Sensitivity analyses using the leave-one-out method are inappropriate. The authors should perform meaningful sensitivity analyses ie. excluding studies with a high risk of bias.

Response:We appreciate your constructive comments and suggestions. We have revised our manuscript accordingly and addressed each point as follows:We agree with the reviewer that the leave-one-out method is not a robust way to assess the sensitivity of our meta-analysis results. Therefore, we have performed additional sensitivity analyses by excluding one with a high risk of bias. We assessed the quality for each study using the Newcastle-Ottawa Quality Assessment Scale.Based on this criterion, we excluded Joh et al. study with a score of 6 on the NOS scale from our meta-analysis: which deified as moderate quality. The result of the sensitivity analysis showed that the exclusion of this study did not change the direction or significance of the pooled HR for any outcome.

7. Egger's test was not designed to explore publication bias in meta-analyses with less than 10 studies.

Response: Thank you for your comment. We appreciate your interest in our study and your constructive feedback. We agree that Egger's test is not suitable for meta-analyses with less than 10 studies, as it may have low power and high false-positive rate. Therefore, we have revised our manuscript to remove the use of Egger's test. We have also added a limitation section to acknowledge the small number of studies included in our meta-analysis and the possibility of publication bias affecting our results. We hope that these changes address your concern and improve the quality of our manuscript.

8. The source of heterogeneity was insufficiently discussed.

Response:Thank you for your comment. We appreciate your interest in our study and your constructive feedback. We agree that the source of heterogeneity is an important issue to address, and we have revised our discussion section accordingly. Here is our response:

We acknowledge that there was substantial heterogeneity across the studied arms for the association between atopic dermatitis and cognitive impairment, especially for Alzheimer’s disease and vascular dementia. We conducted subgroup analyses by region and study design to explore the potential sources of heterogeneity, and we found some differences in the results. For example, we found that the association between atopic dermatitis and all-cause dementia was significant in Europe but not in Asia, and that the association between atopic dermatitis and Alzheimer’s disease-related dementia was significant in prospective cohort studies but not in non-prospective cohort studies. These findings suggest that there may be some regional and methodological factors that influence the relationship between atopic dermatitis and cognitive impairment.

However, we also recognize that there may be other sources of heterogeneity that we could not account for in our meta-analysis, such as the definition and diagnosis of atopic dermatitis, the duration and severity of atopic dermatitis, the presence of comorbidities and confounding factors, the measurement and assessment of cognitive impairment, and the follow-up period and attrition rate of the studies. These factors may vary across different populations and settings, and may affect the risk of developing cognitive impairment among individuals with atopic dermatitis. Therefore, we have added a paragraph in our discussion section to acknowledge these limitations and to suggest future research directions to address them. We hope that this revision will satisfy your concern and improve the quality of our manuscript. Thank you for your valuable suggestion.

9. Several grammatical errors were present.

Response:Thank you for your comment. We appreciate your interest in our study and your constructive feedback. We apologize for the grammatical errors in our manuscript, which may have affected the readability and clarity of our writing. We have revised our manuscript to correct these errors and improve the quality of our language. We have also used a professional editing service to proofread and polish our manuscript. We hope that these changes address your concern and improve the quality of our manuscript.

Reviewer #2: The study question is clear and well defined, looking at the association between atopic dermatitis and cognitive dysfunction. Three databases were searched, Embase, Pubmed and Web of Science.

1. While the study aims to identify the potential mechanisms underlying the association between AD and cognitive dysfunction, the methods section does not describe any specific approaches for exploring these mechanisms, such as analyzing biomarkers or conducting neuroimaging studies. The authors may want to consider incorporating such approaches to gain a better understanding of the underlying potential mechanisms if they are going to describe it as one of the the aims.

Response:Thank you for your comment. We appreciate your interest in our study and your constructive feedback. However, we would like to clarify that exploring the potential mechanisms underlying the association between AD and cognitive dysfunction is not our main goal in this paper. Our primary aim is to conduct a systematic review and meta-analysis of the existing literature on the epidemiological evidence of this association, and to assess the quality and consistency of the studies. We acknowledge that investigating the biological and neurological pathways that may link AD and cognitive dysfunction is an important and interesting topic, but it is beyond the scope of our current paper. Therefore, we have not included any specific approaches for exploring these mechanisms, such as analyzing biomarkers or conducting neuroimaging studies, in our methods section. We hope that you understand our rationale and respect our focus on the epidemiological aspect of this research question. We suggest that future studies may address the mechanistic aspect of this question using more advanced and comprehensive methods.

2. More can be written on how this contributes to utility in clinical practice. More elaboration on the implications for the "prevention, diagnosis, and treatment of cognitive dysfunction in patients with AD".

Response:Thank you for your comment. We appreciate your interest in our study and your constructive feedback. We agree that we should provide more details on how our study contributes to utility in clinical practice and what are the implications for the prevention, diagnosis, and treatment of cognitive dysfunction in patients with AD. Therefore, we have revised our manuscript to include the following paragraphs in the discussion section

3.Please kindly explain why the study Shang et al was used in this study since I could not find mentioning atopic dermatitis in the article.

Response:Thank you for your comment. We apologize for the confusion caused by the incorrect citation. We have corrected the reference to the paper by Shang et al. (2022) that investigated the association of a wide range of chronic diseases, including atopic dermatitis, with subsequent risk of dementia in community-dwelling adults.

*Shang X, Zhu Z, Zhang X, et al. Association of a wide range of chronic diseases and apolipoprotein E4 genotype with subsequent risk of dementia in community-dwelling adults: A retrospective cohort study[J]. EClinicalMedicine, 2022, 45.*

4.The selected articles cover studies from United Kingdom, Taiwan, South Korea and Sweden. It should be mentioned that it may not be representative of all ethnicities and more focused on those in these countries. The "European" population mentioned in the study would come from these countries as well as the "Asian" analysed. It could be mentioned as a limitation and how it could further expand in future studies such as those in North America, other countries in Asian, Africa or Middle Eastern countries, if comparison in the results across ethnicities were to become more accurate and generalizable.

Response:We thank your constructive comments and suggestions. We agree that the selected articles may not be representative of all ethnicities and that the analysis of the European and Asian populations could be more specific. We have revised the manuscript accordingly and added the following paragraph to the discussion section:

*“Second, The majority of the studies were conducted in the United Kingdom, Taiwan, South Korea, and Sweden, which may not reflect the prevalence and impact of atopic dermatitis and cognitive dysfunction in other regions or countries. Future studies should include more diverse and representative samples from different ethnicities and regions, to improve the generalizability and accuracy of the results across different populations. ”*

Reviewer #3: This is an interesting study that focused on middle-aged and older adults and examined the association between atopic dermatitis and the risk of cognitive dysfunction.

1. The methodology and presentation of the authors' work are commendable, but establishing a causal relationship requires an empirical association, temporal priority of the independent variable, and non-spuriousness. Based on the available evidence and current data, it is challenging to definitively conclude that atopic dermatitis increases the risk of cognitive dysfunction.

Response:Thank you for your comment. We appreciate your interest in our study and your constructive feedback. We agree that establishing a causal relationship between atopic dermatitis and cognitive dysfunction requires an empirical association, temporal priority of the independent variable, and non-spuriousness. We acknowledge that our study is limited by the observational nature of the included studies, which may be subject to residual confounding and reverse causation. Therefore, we did not claim that atopic dermatitis causes cognitive dysfunction, but rather that it is associated with an increased risk of cognitive dysfunction.

2.Are there any limitations regarding language when searching and selecting studies?

Response:We appreciate your feedback on our search strategy. We acknowledge that we did not specify the language criterion for our literature search in the manuscript. We have now added a sentence to the Methods section, stating that we only included studies that were published in English. We apologize for this oversight and hope that this clarification addresses your concern.

3.Authors should include detailed annotations that define each indicator used in the Newcastle-Ottawa Scale in Table S4.

Response:We thank the reviewer for their suggestion. In order to provide more clarity on the quality assessment of the included studies, we have added detailed annotations for each indicator used in the Newcastle-Ottawa Scale in Table S4. The annotations explain the criteria for assigning stars to each indicator. We hope that this addition will enhance the transparency and rigor of our systematic review.

4.Figure 3 is challenging to read, improvements should be made to enhance its readability.

Response:Thank you for your valuable comments and suggestions. We have revised Figure 3 to make it more readable and clear. We hope that these changes will improve the quality and clarity of our manuscript.

5.P-values can never be 0. Please review the results in Figure 1, Supplementary Tables 4, 5, and 6 to ensure accurate reporting. If the exact p-value is less than 0.001, it is appropriate to state "p < 0.001". Otherwise, report the exact p-values.

Response:We thank the reviewer for their careful reading and constructive feedback. We have revised the manuscript to address the issue of p-values reporting. We have checked the results in Figure 1, Supplementary Tables 4, 5, and 6 and corrected any instances where p-values were reported as 0. We have also followed the reviewer's suggestion to state "p < 0.001" when the exact p-value is less than 0.001. We hope that these changes improve the clarity and accuracy of our findings.

6. The results from Figure 2 demonstrate high heterogeneity and imprecision. However, the potential causes of heterogeneity could not be fully explored.

Response:We thank your constructive comments. We agree that the results from Figure 2 show high heterogeneity and imprecision among the studies included in the meta-analysis. We have added a paragraph in the discussion section to address the possible sources of heterogeneity. We have also performed a sensitivity analysis to assess the robustness of our findings by excluding studies with a high risk of bias . The revised paragraph is as follows:

*“Fourth, Several factors may contribute to the heterogeneity observed in our meta-analysis. For example, the studies varied in their design, such as prospective cohort, retrospective cohort, and cross-sectional studies, which may have different biases and confounding effects. Furthermore, the studies included different populations of middle-aged and older adults, with different geographic locations, ethnicities, and socioeconomic status. In addition, the studies used different definitions of atopic dermatitis and cognitive dysfunction, which may affect the validity and comparability of the results. ”*

We hope this response addresses your concern satisfactorily. Thank you again for your time and effort in reviewing our manuscript.
