# Supplementary material for: Atopic dermatitis and cognitive dysfunction in middle-aged and older adults: A systematic review and meta-analysis
Source: PLoS One. 2023 Oct 25;18(10):e0292987. doi: 10.1371/journal.pone.0292987 (PMC10599501; doi:10.1371/journal.pone.0292987)
Supplement: Attachment — Submitted filename: Response to Reviewers.docx [file pone.0292987.s012.docx]

Dear editor and reviewers,

Thank you for your letter dated September 12, 2023. On behalf of my colleagues, I am herewith submitting the revised manuscript (PONE-D-23-12015) entitled “Atopic dermatitis and cognitive dysfunction in middle-aged and older adults: A systematic review and meta-analysis” for consideration of publication in Plos one. We would like to thank the editor and reviewers’ work devoted to our manuscript and we are very grateful for their valuable suggestions. We have considered the comments carefully and have made revisions (highlighted in red in the revised manuscript with track changes ) which we hope meet with approval.

Journal requirements:

1. Please review your reference list to ensure that it is complete and correct. If you have cited papers that have been retracted, please include the rationale for doing so in the manuscript text, or remove these references and replace them with relevant current references. Any changes to the reference list should be mentioned in the rebuttal letter that accompanies your revised manuscript. If you need to cite a retracted article, indicate the article’s retracted status in the References list and also include a citation and full reference for the retraction notice.

Response: We thank the reviewer for their valuable comments and suggestions.we have checked our reference list.

Comments to the Author:

Reviewer #2: The study has been well conducted with a few improvements that can be made that can make it a strong publication.

1. The inclusion criteria could be better defined, especially the definitions for cognitive dysfunction and middle-aged/older adults. For example, the age range or define the age cutoffs. For example for cognitive dysfunction, * you can refer to standard criteria or definitions used in published guidelines, such as the DSM-5 criteria for mild neurocognitive disorder or major neurocognitive disorder, or require that cognitive dysfunction was assessed through neuropsychological testing or clinical diagnosis, or specify the required threshold on cognitive screening tests like the Mini-Mental State Exam (MMSE) or Montreal Cognitive Assessment (MoCA).

Response: Thank you for your valuable feedback. We have revised our inclusion criteria as follows:We defined cognitive dysfunction as a category of cognitive impairment diseases that met the DSM-5 criteria for various subtypes of cognitive disorders, including all-cause dementia, Alzheimer's disease dementia, vascular dementia, mild cognitive impairment[1]. We defined middle-aged adults as those between 45 and 59 years old, while older adults as those aged 60 years and above[2].

References

[1]Majer R, Simon V, Csiba L, et al. Behavioural and psychological symptoms in neurocognitive disorders: Specific patterns in dementia subtypes[J]. Open Medicine, 2019, 14(1): 307-316.

[2] World Health Organization. (2020). Ageing and health.

2. Minor grammatical and language errors can be changed, such as "a thorough search of electronic databases". Or "Despite these findings, there was still a lack of consensus regarding the nature of the relationship between AD and cognitive dysfunction."

Response: We thank for your constructive comments and suggestions. We have corrected the minor grammatical and language errors throughout the manuscript. For example, we have changed "a thorough search of electronic databases" to "a comprehensive search of electronic databases" in the Abstract section, and "Despite these findings, there was still a lack of consensus regarding the nature of the relationship between AD and cognitive dysfunction." to "However, these findings did not resolve the controversy over the molecular mechanism between AD and cognitive dysfunction." in the Introduction section.

3.More attention can be given to the clinical and public health implications of the findings.

Response: Thank you for your constructive comments and suggestions on our manuscript. We have revised our manuscript according to your requirements and added more discussion on the clinical and public health implications of our findings.We also acknowledged the limitations of our study and suggested directions for future research.

Reviewer #3: Most comments have been addressed well; however, I can't find your updated version of figures and tables.

Response: We apologize for the inconvenience caused by the missing figures and tables in our previous submission. We have updated our manuscript with the revised and added figures and tables, which are now available in the supplementary materials. Thank you for your valuable feedback and suggestions.
